# Statin Drugs Are Associated With Response to Immune Checkpoint Blockade in Recurrent/Metastatic Head and Neck Cancer

**DOI:** 10.1002/cam4.70718

**Published:** 2025-03-07

**Authors:** Tyler J. Kristoff, Sean Evans, Pranay Nayi, Marin Abousaud, Subir Goyal, Yuan Liu, Dong Shin, Conor E. Steuer, Nabil F. Saba, Nicole C. Schmitt

**Affiliations:** ^1^ Department of Hematology and Medical Oncology Emory University Atlanta Georgia USA; ^2^ Winship Cancer Institute, Emory University Atlanta Georgia USA; ^3^ Children's Healthcare of Atlanta Atlanta Georgia USA; ^4^ Astellas Pharma Global Development Inc Northbrook Illinois USA; ^5^ Department of Biostatistics and Bioinformatics Rollins School of Public Health of Emory University Atlanta Georgia USA; ^6^ Department of Otolaryngology – Head and Neck Surgery Emory University Atlanta Georgia USA

**Keywords:** head and neck cancer, immune checkpoint blockade, immunotherapy, PD‐1, PD‐L1, squamous cell carcinoma, statins

## Abstract

**Background:**

Statin drugs, frequently used to treat hyperlipidemia, are associated with improved survival outcomes in multiple solid tumor types, including head and neck squamous cell carcinoma (HNSCC). Preclinical studies suggest that manipulation of cholesterol with statins and other agents can enhance the function of multiple components involved in anti‐tumor immune responses. Retrospective studies in other solid tumor types suggest that statin therapy is associated with improved responses to immune checkpoint blockade (ICB), but this has not yet been investigated in HNSCC.

**Methods:**

Pharmacy records were searched for patients with recurrent/metastatic HNSCC treated at our institution with pembrolizumab or nivolumab from 2015 to 2022. Patients who received less than 3 doses of ICB were excluded. Univariate and multivariate analyses were performed to determine the association between statin use and objective response, progression‐free survival (PFS) and overall survival (OS).

**Results:**

A total of 158 patients were included. Statins were significantly associated with objective response; the strongest associations were seen with rosuvastatin and lovastatin. On multivariate analyses, statins were independently associated with objective response but not with PFS or OS.

**Conclusions:**

Statin therapy appears to be an independent predictor of response to ICB in HNSCC. Larger, prospective studies are needed to determine whether specific statin drugs can improve survival outcomes in ICB‐treated patients.

## Graphical abstract

Cholesterol manipulation has recently been associated with enhanced responses to immunotherapy. Review of clinical outcomes among patients treated with immune checkpoint blockade for head and neck cancer at our institution shows that statin use was independently associated with an objective response, but no significant increase in survival.

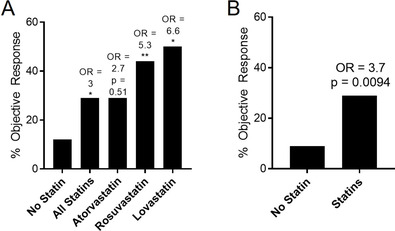

## 1 Introduction

Approximately half of patients with head and neck squamous cell carcinoma (HNSCC) who are treated with standard therapy will develop recurrent and/or metastatic disease [1]. Anti‐PD‐1 immune checkpoint blockade (ICB) is FDA‐approved for use alone or in combination with chemotherapy in the first‐line recurrent/metastatic setting based on results of the KEYNOTE‐048 trial [2]. However, only a fraction of patients (18%) typically respond [2]. Clinical strategies to enhance responses to anti‐PD‐1 ICB without excessive cost or toxicity are needed.

Statin drugs, which are HMG‐CoA reductase inhibitors, are frequently used for the treatment of hyperlipidemia. It is well known that statin drugs may also have anti‐cancer, anti‐inflammatory, and other pleiotropic effects [3, 4]. Prior retrospective studies in several cancer types have shown that statin use is associated with improved outcomes [5, 6, 7, 8, 9, 10]. In head and neck cancer patients, statin use is associated with improved survival [9, 10], lower incidence of cisplatin‐induced hearing loss [11], and improvement of radiation‐induced fibrosis [12], all by mechanisms that are not well understood. Although the potential benefit of statins for recurrent/metastatic head and neck cancer treated with anti‐PD‐1 ICB has not yet been investigated, studies in other cancer types suggest that statins are associated with increased rates of response to ICB [13, 14]. However, the off‐target effects of different statins can be highly variable [11, 13], and studies on the effects of specific statin drugs are lacking.

In preclinical models of oral cancer, we showed that statin drugs, particularly lovastatin and simvastatin, can enhance responses to anti‐PD‐1 ICB, with increased T cell activation in the tumor draining lymph node [15]. Lovastatin + anti‐PD‐1 ICB was particularly effective, resulting in tumor rejection in 30% of mice bearing MOC1 tumors and durable immunologic memory. In the present study, we reviewed cases of recurrent/metastatic HNSCC treated at our institution with at least 3 doses of nivolumab or pembrolizumab to assess whether statin therapy is associated with a higher incidence of response to these drugs and/or improved survival outcomes. We also aimed to determine whether associations with immunotherapy response differ among specific statin drugs.

## 2 Methods

### 2.1 Patient Characteristics and Records Search

Pharmacy records from 2015 to 2022 were queried for patients with pathologically confirmed HNSCC treated with nivolumab or pembrolizumab. Patients with salivary tumors or who received fewer than three doses of ICB were excluded. Patients who participated in an open‐label, phase II trial of pembrolizumab and cabozantinib were also excluded, as this combination was associated with a high response rate [16]. Clinical information was collected, including demographics, clinicopathologic characteristics, including disease site and p16 status (as a surrogate for disease driven by human papillomavirus), combined positive score for PD‐L1 staining, body mass index (BMI), dates and duration of therapy, ECOG performance status (PS), initiation of radiation and/or chemotherapy during ICB, and immune‐related adverse events (irAEs). For any patient taking a statin drug at the time of immunotherapy, the specific drug and dose were recorded. Response was determined via retrospective review of clinical notes and imaging reports and was defined as a partial or complete response during the first 6 months of therapy. Progression‐free survival (PFS) was defined as the time from initiation of immunotherapy to disease progression, and overall survival (OS) was defined as the time from initiation of immunotherapy to death from any cause.

### 2.2 Statistical Analyses

Patient characteristics were summarized using descriptive statistics. Baseline and clinical characteristics between the two treatment groups (anti‐PD‐1 ICB alone versus anti‐PD‐1 ICB plus statin) were compared using Chi‐square or Fisher's Exact tests for categorical covariates and the ANOVA or Kruskal‐Wallis tests for numerical covariates. The Kaplan–Meier method was used to generate OS and PFS curves, and survival curves were compared between the two treatment cohorts using the log‐rank test. Univariate (UVA) and multivariable (MVA) analyses were performed to assess factors associated with OS and PFS using the Cox proportional hazards model. UVA and MVA using logistic regression were employed to estimate odds ratios (ORs) for objective response. Clinically relevant covariates were included in the multivariable models. Specifically, ECOG performance status, initiation of chemotherapy during immunotherapy, and concomitant statin use were included. A *p*‐value of < 0.05 was considered statistically significant for all analyses. All analyses were conducted using SAS Version 9.4 and SAS macros developed by the Biostatistics Shared Resource at Winship Cancer Institute [17].

### 2.3 Study Approval

This study was approved by the Institutional Review Board of Emory University (Study #00004222). Informed consent was not obtained from individual patients.

## 3 Results

### 3.1 Statin Use Is Associated With Response to Nivolumab and Pembrolizumab

**FIGURE 1:**
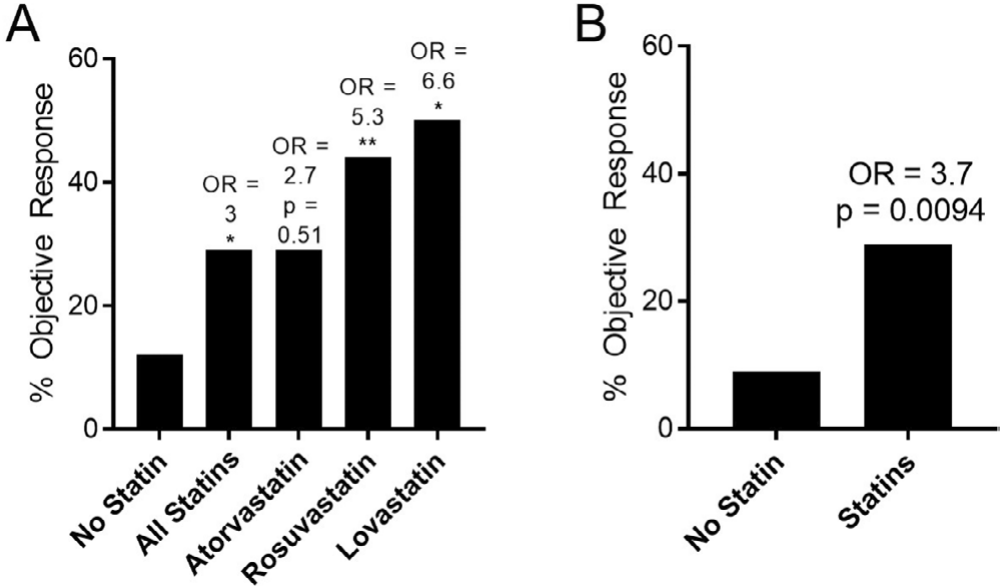
Percent objective response with and without statin therapy. (A) Response according to therapy with specific statins versus all or no statins. (B) Response according to statin use among patients not treated with chemotherapy during immune checkpoint blockade. OR, odds ratio. *p < 0.05, **p < 0.01 versus no statin therapy.

Patient demographics and disease characteristics are listed in Table 1. Median follow‐up was 28.32 months (95% confidence interval, 23.76–36.72 months). The majority of patients were male and had good performance status. A total of 44 patients (27.8%) were taking statins while on treatment with pembrolizumab or nivolumab; 24 of these patients (54%) were taking atorvastatin, which was by far the most common statin drug. When comparing patients according to statin use, the patients taking statins had a higher mean BMI and were more likely to be male (Table 2). The patients taking statins appeared to have a better performance status and were more often treated with chemotherapy while on ICB, although these results did not quite reach statistical significance (*p* = 0.069 and 0.067, respectively). Patients taking statins were more likely to demonstrate an objective response to ICB (Tables 2 and 3, Figure 1), especially patients taking lovastatin or rosuvastatin (Figure 1A). The few patients (*n* = 7) taking simvastatin or pravastatin all showed either stable or progressive disease. Since the use of platinum chemotherapy was more common in the statin group and can increase the chance of an objective response, we also performed a separate analysis with exclusion of patients receiving chemotherapy (Figure 1B). Although we did not have adequate statistical power to detect differences with individual statin drugs, patients treated with statins still had a higher incidence of response even in the absence of chemotherapy (odds ratio 3.7, *p* = 0.0094). On multivariate analysis, statin therapy was found to be an independent predictor of objective response to ICB (OR = 2.5, *p* = 0.045; Table 4).

**TABLE 1:** Demographic, clinicopathologic, and treatment data for included patients.

	Total *n* = 158
Patient demographics	
Age, mean (SD)	64 (12)
Sex	
Male, *n* (%)	121 (76.6)
Female, *n* (%)	37 (23.4)
Race	
White, *n* (%)	104 (68)
Black, *n* (%)	32 (20.9)
Asian, *n* (%)	17 (11.1)
Clinicopathologic data	
Tumor Subsite	
Nasopharynx, *n* (%)	11 (7.0)
Sinonasal, *n* (%)	7 (4.4)
Oral cavity, *n* (%)	47 (29.7)
Larynx, *n* (%)	14 (8.9)
Hypopharynx, *n* (%)	16 (10.1)
Other (cutaneous), *n* %	1 (0.6)
Oropharynx, *n* (%)	62 (39.2)
P16‐negative, *n* (%)	17 (27.4)
P16‐positive, *n* (%)	34 (54.8)
Unknown, *n* (%)	11 (17.7)
Combined positive score (CPS)	
< 1, *n* (%)	3 (1.9)
≥ 1 and < 20, *n* (%)	34 (21.5)
≥ 20, *n* (%)	20 (12.7)
Unknown	101 (63.9)
ECOG performance status	
0, *n* (%)	13 (8.2)
1, *n* (%)	114 (72.2)
2, *n* (%)	26 (16.5)
3, *n* (%)	1 (0.6)
Not recorded, *n* (%)	14 (8.9)

**TABLE 2:** Descriptive statistics by statin therapy.

Covariate	Total *N* = 158 (100%)	Statin therapy		*p* ^b^
		No *N* = 114 (72.2%)	Yes *N* = 44 (27.8%)	
Gender, *N* (%)				**0.026**
Female	37 (23.4)	32 (28.1)	5 (11.4)	
Male	121 (76.6)	82 (71.9)	39 (88.6)	
Race, *N* (%)				0.661
White	104 (68)	77 (70)	27 (62.8)	
Black	32 (20.9)	22 (20)	10 (23.3)	
Other	17 (11.1)	11 (10)	6 (14)	
Drug prescribed, *N* (%)				0.137
Pembrolizumab	120 (75.9)	83 (72.8)	37 (84.1)	
Nivolumab	38 (24.1)	31 (27.2)	7 (15.9)	
ECOG PS at baseline, *N* (%)				0.069
0–1	117 (81.3)	79 (77.5)	38 (90.5)	
2–3	27 (18.8)	23 (22.5)	4 (9.5)	
irAEs, *N* (%)				0.533
No	96 (63.6)	67 (62)	29 (67.4)	
Yes	55 (36.4)	41 (38)	14 (32.6)	
Baseline BMI				**0.021** ^a^
Total *N*	151	109	42	
Mean (std dev)	23.5 (4.6)	23 (4.7)	24.8 (4.3)	
Median (Q1–Q3)	22.9 (20.4–26)	22.5 (20–24.9)	24.2 (21.2–28.1)	
Min—max	14.2–37.5	14.2–37.5	17–34.6	
Initiation of chemotherapy, *N* (%)				0.0667
No	120 (75.9)	91 (79.8)	29 (65.9)	
Yes	38 (24.1)	23 (20.2)	15 (34.1)	
Initiation of radiation, *N* (%)				0.806
No	145 (94.3)	105 (92.1)	40 (90.9)	
Yes	13 (5.7)	9 (7.9)	4 (9.1)	
Number of cycles of ICB				0.136^a^
Total *N*	150	110	40	
Mean (std dev)	8.8 (8.6)	8.1 (7)	10.6 (11.9)	
Median (Q1–Q3)	6 (4–10)	6 (4–10)	6.5 (5–11)	
Min–Max	3–70	3–46	3–70	

^a^ A non‐parametric test (Kruskal‐Wallis or Fisher's exact test) is applied.

^b^ The *p*‐value is calculated by either parametric (ANOVA, Chi‐squared) or non‐parametric (Kruskal‐Wallis, Fisher's exact) tests, where appropriate, based on the normality test of data distribution and the sample size. Bold font indicates statistically significant values.

**TABLE 3:** Univariate logistic regression for objective response.

Covariate	Level	*N*	Objective response = yes		
			Odds ratio (95% CI)	OR *p*	Overall *p*
Statin therapy	Yes	44	3.00 (1.27–7.05)	**0.012**	**0.012**
	No	114	—	—	
Race	Black	32	0.78 (0.27–2.27)	0.646	0.718
	Other	17	0.56 (0.12–2.65)	0.465	
	White	104	—	—	
Gender	Female	37	0.92 (0.34–2.49)	0.872	0.872
	Male	121	—	—	
Drug prescribed	Pembrolizumab	120	1.48 (0.52–4.22)	0.463	0.463
	Nivolumab	38	—	—	
ECOG baseline	2–3	27	1.04 (0.35–3.06)	0.944	0.944
	0–1	117	—	—	
irAEs	Yes	55	1.81 (0.78–4.21)	0.166	0.166
	No	96	—	—	
Initiation of chemotherapy	Yes	38	2.16 (0.89–5.25)	0.088	0.088
	No	120	—	—	

*Note:* Bold font indicates statistically significant values.

**TABLE 4:** Multivariate logistic regression for objective response.

Covariate	Level	*N*	Objective response = yes		
			Odds Ratio (95% CI)	OR *p*	Overall *p*
Statin therapy	Yes	42	2.50 (1.02–6.15)	0.045	**0.045**
	No	102	—	—	
ECOG baseline	2–3	27	1.38 (0.44–4.27)	0.581	0.581
	0–1	117	—	—	
Initiation of chemotherapy	Yes	37	1.61 (0.62–4.14)	0.325	0.325
	No	107	—	—	

*Note:* Number of observations in the original data set = 158. Number of observations used = 144. Bold font indicates statistically significant values.

### 3.2 Statin Use Is Not Independently Associated With Improved Survival

**FIGURE 2:**
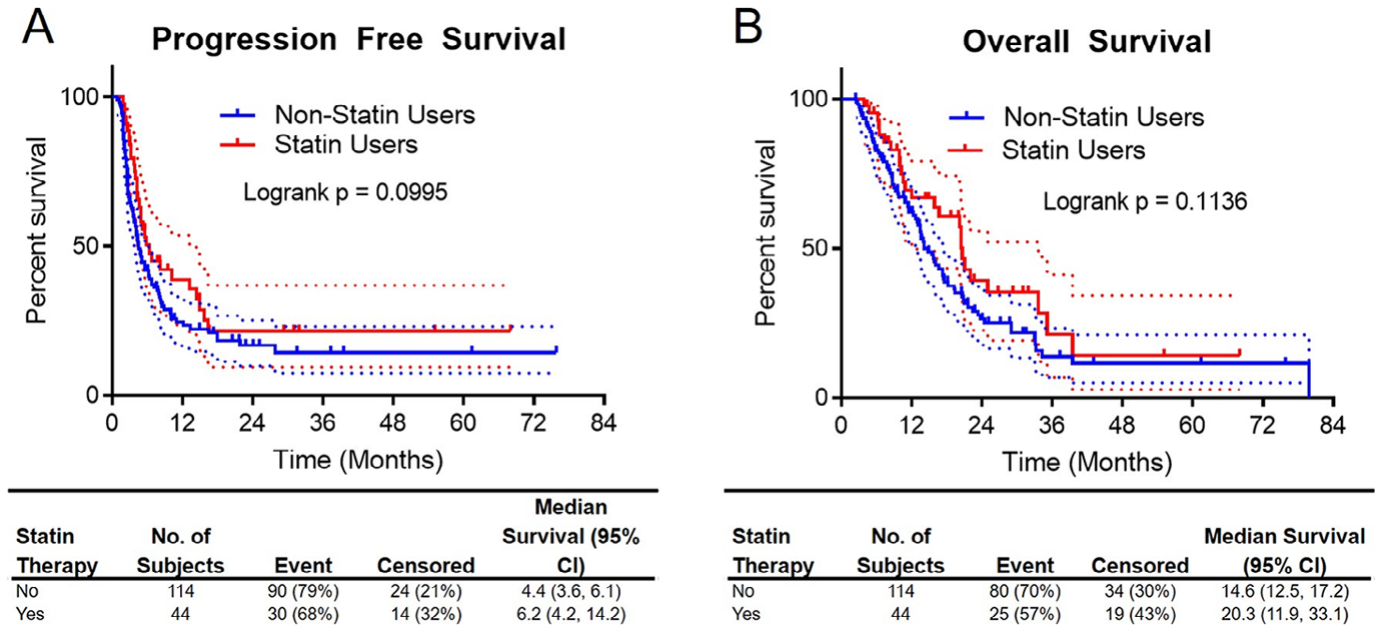
Progression‐free (A) and overall survival (B) were not significantly different based on statin therapy.

We next investigated whether the improved responses with statin drugs lead to improvements in PFS and OS. When comparing all statin users to statin non‐users, there were modest improvements in survival that did not quite reach statistical significance (Figure 2, Tables 5, 6, 7, 8). On univariate analyses, only the number of ICB cycles was associated with significantly improved PFS. On multivariate analysis, statin therapy did not quite reach statistical significance as an independent predictor of PFS (HR = 0.72, *p* = 0.134). Not surprisingly, a better ECOG performance status and a greater number of ICB cycles were associated with improved overall survival.

**TABLE 5:** Univariate association with progression free survival.

Covariate	Level	*N*	Progression (months)		
			Hazard ratio (95% CI)	HR *p*	Log‐rank *p*
Statin therapy	Yes	44	0.71 (0.47–1.07)	0.105	0.100
	No	114	—	—	
Race	Black	32	1.11 (0.69–1.76)	0.671	0.413
	Other	17	1.44 (0.83–2.51)	0.194	
	White	104	—	—	
Gender	Female	37	1.13 (0.74–1.71)	0.574	0.570
	Male	121	—	—	
Drug prescribed	Pembrolizumab	120	1.01 (0.67–1.52)	0.956	0.955
	Nivolumab	38	—	—	
ECOG baseline	2–3	27	1.53 (0.97–2.42)	0.065	0.060
	0–1	117	—	—	
irAEs	Yes	55	0.93 (0.63–1.36)	0.701	0.698
	No	96	—	—	
Initiation of chemotherapy	Yes	38	0.84 (0.55–1.29)	0.433	0.428
	No	120	—	—	
Baseline BMI		151	0.97 (0.93–1.01)	0.176	—
Number of cycles of ICB		150	0.86 (0.82–0.90)	**< 0.001**	—

*Note:* Bold font indicates statistically significant values.

Abbreviations: ICB, immune checkpoint blockade; irAEs, immune related adverse events.

**TABLE 6:** MVA Cox proportional hazard model for progression free survival.

Covariate	Level	Progression (mths)		
		Hazard ratio (95% CI)	HR *p*	Overall *p*
Statin therapy	Yes	0.72 (0.46–1.11)	0.134	0.134
	No	—	—	
ECOG baseline	2–3	1.48 (0.94–2.34)	0.091	0.091
	0–1	—	—	
Initiation of chemotherapy	Yes	0.90 (0.57–1.40)	0.628	0.628
	No	—	—	

*Note:* Number of observations in the original data set = 158. Number of observations used = 144.

**TABLE 7:** Univariate association with overall survival.

Covariate	Level	*N*	OS (months)		
			Hazard ratio (95% CI)	HR *p*	Log‐rank *p*
Statin therapy	Yes	44	0.70 (0.44–1.09)	0.117	0.114
	No	114	—	—	
Race	Black	32	1.21 (0.75–1.97)	0.432	0.636
	Other	17	0.86 (0.41–1.80)	0.695	
	White	104	—	—	
Gender	Female	37	1.21 (0.77–1.88)	0.408	0.405
	Male	121	—	—	
Drug prescribed	Pembrolizumab	120	1.24 (0.79–1.95)	0.343	0.340
	Nivolumab	38	—	—	
ECOG baseline	2–3	27	2.27 (1.41–3.64)	**< 0.001**	**< 0.001**
	0–1	117	—	—	
irAEs	Yes	55	1.09 (0.73–1.65)	0.663	0.662
	No	96	—	—	
Initiation of chemotherapy	Yes	38	0.67 (0.40–1.11)	0.119	0.115
	No	120	—	—	
Baseline BMI		151	0.96 (0.91–1.00)	**0.048**	—
Number of cycles of ICB		150	0.88 (0.84–0.92)	**< 0.001**	—

*Note:* Bold font indicates statistically significant values.

Abbreviations: BMI, body mass index; ICB, immune checkpoint blockade; irAEs, immune related adverse events.

**TABLE 8:** MVA Cox proportional hazard model for overall survival.

Covariate	Level	OS (mths)		
		Hazard ratio (95% CI)	HR *p*	Overall *p*
Statin therapy	Yes	0.78 (0.49–1.24)	0.292	0.292
	No	—	—	
ECOG baseline	2–3	2.27 (1.41–3.65)	**< 0.001**	**< 0.001**
	0–1	—	—	
Initiation of chemotherapy	Yes	0.75 (0.45–1.27)	0.285	0.285
	No	—	—	

*Note:* Number of observations in the original data set = 158. Number of observations used = 144. Bold font indicates statistically significant values.

## 4 Discussion

Our data suggest that the use of statins may improve responses to ICB in recurrent/metastatic HNSCC. However, in this relatively small cohort, wherein only a small proportion of patients were taking statins (27.8%), we did not see a statistically significant improvement in survival. Similar results were seen in a large, multi‐institutional cohort of patients with renal cell carcinoma, melanoma, and non‐small cell lung cancer treated with anti‐PD‐1 ICB: statin use was associated with a significant improvement in objective response (odds ratio 1.6) but was not associated with improved survival [14]. Data on individual statin drugs were not shown in that study.

Numerous retrospective studies have shown improved survival in patients with solid tumors who are taking statins [3, 5, 6, 7, 8, 9, 10, 18], although the mechanisms are not entirely understood. There are multiple large, retrospective studies demonstrating that patients taking statins upon diagnosis of HNSCC have improved survival outcomes [9, 10, 18]. Preclinical studies suggest that statins have direct anti‐proliferative effects on tumor cells [19, 20]. Statin drugs may show additive or synergistic activity with chemotherapy [21, 22, 23], and one preclinical study showed that this may be in part due to the ability of statins to abrogate chemotherapy‐induced upregulation of PD‐L1 on tumor cells [21].

Manipulation of cholesterol may also indirectly impact tumor growth by enhancing the anti‐tumor immune response [15, 19, 22, 23, 24, 25, 26, 27, 28, 29, 30, 31, 32, 33]. Our prior studies in preclinical models of HNSCC suggest that all statins have anti‐proliferative effects, but lovastatin and simvastatin specifically can also enhance T‐cell activation and M1 macrophage polarization [15]. In a mouse model of oral cancer, the combined use of anti‐PD‐1 ICB and lovastatin resulted in tumor rejection and immunologic memory in 30% of animals [15]. The mechanisms by which statins directly enhance the function of CD8^+^ T cells are unclear. However, it has been shown that cholesterol in the T cell membrane can impact T cell receptor (TCR) activation and downstream signaling [28]. Our recent unpublished preclinical work with collaborators at the Georgia Institute of Technology suggests that the affinity of the TCR for its cognate peptide–MHC, and the force of the resulting bonds between them, are impaired in the tumor microenvironment but can be restored with statin therapy [34].

The off‐target effects of different statin drugs can vary dramatically [11], and it is plausible that the mixed results on statin drugs seen in many studies to date can be attributed in part to these differences in the effects of individual statins. Atorvastatin, the most commonly used statin in the United States, was associated with a modest improvement in response in the present study. In contrast, lovastatin, which was the most promising drug in our preclinical studies, had an odds ratio of 6.6 for objective response. It is thus plausible that if all patients were taking specific drugs such as lovastatin or rosuvastatin, we might detect an improvement in survival. However, in a ‘real world’ cohort such as this one where multiple statins are in use for hyperlipidemia, the effects of specific, particularly effective statin drugs may be obscured.

In addition to its retrospective nature, the present study has additional limitations. The population of patients was relatively heterogeneous. The combined positive score (CPS) for PD‐L1 staining is a known prognostic factor in response to anti‐PD‐1 ICB, but this was not established until after the KEYNOTE‐048 trial (published in 2019). Because many of the patients in our cohort were treated years earlier, we do not have CPS data for much of our cohort. Lastly, a large number of patients were also treated with chemotherapy while on treatment with ICB, limiting our ability to detect improved responses with individual statins in the absence of other treatments. However, when we included concomitant chemotherapy in multivariable models, it was not independently associated with improved response. We also do not have biospecimens from these patients, which would allow us to explore potential mechanisms by which statins improve anti‐tumor immune responses. However, we recently opened a clinical trial of lovastatin and pembrolizumab for patients with recurrent/metastatic HNSCC (NCT06636734) at our institution. Blood and tumor biospecimens from this trial are expected to provide substantial mechanistic insight in the near future. Despite these limitations, our data suggest that the use of specific statins drugs deserves further study as an affordable, well‐tolerated means of improving the proportion of patients with HNSCC who may benefit from immune checkpoint inhibitors.

## Author Contributions

**Tyler J. Kristoff:** conceptualization (equal), data curation (equal), investigation (equal), methodology (equal), writing – original draft (equal), writing – review and editing (equal). **Sean Evans:** data curation (equal), writing – review and editing (equal). **Pranay Nayi:** data curation (equal), writing – review and editing (equal). **Marin Abousaud:** data curation (equal), writing – review and editing (equal). **Subir Goyal:** formal analysis (equal), methodology (equal), writing – original draft (equal), writing – review and editing (equal). **Yuan Liu:** formal analysis (equal), supervision (equal), writing – original draft (equal), writing – review and editing (equal). **Dong Shin:** data curation (supporting), writing – review and editing (equal). **Conor E. Steuer:** data curation (supporting), writing – review and editing (equal). **Nabil F. Saba:** conceptualization (equal), data curation (supporting), supervision (supporting), writing – original draft (equal), writing – review and editing (equal). **Nicole C. Schmitt:** conceptualization (lead), data curation (supporting), formal analysis (equal), funding acquisition (lead), investigation (supporting), methodology (lead), project administration (lead), supervision (lead), writing – original draft (lead), writing – review and editing (lead).

## Disclosure

Schmitt—Consulting/Advisory Board: Regeneron, Geovax, Sensorion. Book Royalties: Plural Publishing. Clinical Trial Funding: Astex Pharmaceuticals.

## Conflicts of Interest

The authors declare no conflicts of interest.

## Data Availability

De‐identified data will be made available for research purposes upon reasonable request.

## References

[1] M. D. Mody , J. W. Rocco , S. S. Yom , R. I. Haddad , and N. F. Saba , “Head and Neck Cancer,” Lancet 398, no. 10318 (2021): 2289–2299. 34562395 10.1016/S0140-6736(21)01550-6

[2] B. Burtness , K. J. Harrington , R. Greil , et al., “Pembrolizumab Alone or With Chemotherapy Versus Cetuximab With Chemotherapy for Recurrent or Metastatic Squamous Cell Carcinoma of the Head and Neck (KEYNOTE‐048): A Randomised, Open‐Label, Phase 3 Study,” Lancet 394, no. 10212 (2019): 1915–1928. 31679945 10.1016/S0140-6736(19)32591-7

[3] J. Longo , J. E. van Leeuwen , M. Elbaz , E. Branchard , and L. Z. Penn , “Statins as Anticancer Agents in the Era of Precision Medicine,” Clinical Cancer Research 26, no. 22 (2020): 5791–5800. 32887721 10.1158/1078-0432.CCR-20-1967

[4] P. F. Zhu , M. X. Wang , Z. L. Chen , and L. Yang , “Targeting the Tumor Microenvironment: A Literature Review of the Novel Anti‐Tumor Mechanism of Statins,” Frontiers in Oncology 11 (2021): 761107. 34858839 10.3389/fonc.2021.761107 PMC8632059

[5] M. K. Nowakowska , X. Lei , M. T. Thompson , et al., “Association of Statin Use With Clinical Outcomes in Patients With Triple‐Negative Breast Cancer,” Cancer 127 (2021): 4142–4150. 34342892 10.1002/cncr.33797 PMC11912801

[6] M. Yuan , S. Han , Y. Jia , et al., “Statins Are Associated With Improved Survival of Patients With Gastric Cancer: A Systematic Review and Meta‐Analysis,” International Journal of Clinical Practice 2022 (2022): 4938539. 35685487 10.1155/2022/4938539 PMC9158792

[7] E. L. Craig , K. H. Stopsack , E. Evergren , et al., “Statins and Prostate Cancer‐Hype or Hope? The Epidemiological Perspective,” Prostate Cancer and Prostatic Diseases 25 (2022): 641–649. 35732821 10.1038/s41391-022-00554-1 PMC9705231

[8] M. Santoni , F. S. M. Monteiro , F. Massari , et al., “Statins and Renal Cell Carcinoma: Antitumor Activity and Influence on Cancer Risk and Survival,” Critical Reviews in Oncology/Hematology 176 (2022): 103731. 35718065 10.1016/j.critrevonc.2022.103731

[9] A. Gupta , W. Stokes , M. Eguchi , et al., “Statin Use Associated With Improved Overall and Cancer Specific Survival in Patients With Head and Neck Cancer,” Oral Oncology 90 (2019): 54–66. 30846177 10.1016/j.oraloncology.2019.01.019 PMC6659746

[10] N. L. Lebo , R. Griffiths , S. Hall , J. Dimitroulakos , and S. Johnson‐Obaseki , “Effect of Statin Use on Oncologic Outcomes in Head and Neck Squamous Cell Carcinoma,” Head & Neck 40, no. 8 (2018): 1697–1706. 29934959 10.1002/hed.25152

[11] K. A. Fernandez , P. Allen , M. Campbell , et al., “Atorvastatin Is Associated With Reduced Cisplatin‐Induced Hearing Loss,” Journal of Clinical Investigation 131, no. 1 (2021): e142616. 33393488 10.1172/JCI142616 PMC7773379

[12] C. Bourgier , A. Auperin , S. Rivera , et al., “Pravastatin Reverses Established Radiation‐Induced Cutaneous and Subcutaneous Fibrosis in Patients With Head and Neck Cancer: Results of the Biology‐Driven Phase 2 Clinical Trial Pravacur,” International Journal of Radiation Oncology, Biology, Physics 104, no. 2 (2019): 365–373. 30776452 10.1016/j.ijrobp.2019.02.024

[13] L. Cantini , F. Pecci , D. P. Hurkmans , et al., “High‐Intensity Statins Are Associated With Improved Clinical Activity of PD‐1 Inhibitors in Malignant Pleural Mesothelioma and Advanced Non‐Small Cell Lung Cancer Patients,” European Journal of Cancer 144 (2021): 41–48. 33326868 10.1016/j.ejca.2020.10.031

[14] A. Cortellini , M. Tucci , V. Adamo , et al., “Integrated Analysis of Concomitant Medications and Oncological Outcomes From PD‐1/PD‐L1 Checkpoint Inhibitors in Clinical Practice,” Journal for Immunotherapy of Cancer 8, no. 2 (2020): e001361. 33154150 10.1136/jitc-2020-001361 PMC7646355

[15] V. Kansal , A. Burnham , B. Kinney , et al., “Statin Drugs Enhance Responses to Immune Checkpoint Blockade in Head and Neck Cancer Models,” Journal for Immunotherapy of Cancer 5 (2023): 5940. 10.1136/jitc-2022-005940 PMC9853267 36650022

[16] N. F. Saba , C. E. Steuer , A. Ekpenyong , et al., “Pembrolizumab and Cabozantinib in Recurrent Metastatic Head and Neck Squamous Cell Carcinoma: A Phase 2 Trial,” Nature Medicine 29, no. 4 (2023): 880–887. 10.1038/s41591-023-02275-x PMC10205145 37012550

[17] Y. Liu , D. C. Nickleach , C. Zhang , J. M. Switchenko , and J. Kowalski , “Carrying out Streamlined Routine Data Analyses With Reports for Observational Studies: Introduction to a Series of Generic SAS ((R)) Macros,” F1000 Research 7 (2018): 1955. 31231506 10.12688/f1000research.16866.1 PMC6567291

[18] K. R. Getz , E. Bellile , K. R. Zarins , et al., “Statin Use and Head and Neck Squamous Cell Carcinoma Outcomes,” International Journal of Cancer 148, no. 10 (2021): 2440–2448. 33320960 10.1002/ijc.33441 PMC8203748

[19] H. R. Jin , J. Wang , Z. J. Wang , et al., “Lipid Metabolic Reprogramming in Tumor Microenvironment: From Mechanisms to Therapeutics,” Journal of Hematology & Oncology 16, no. 1 (2023): 103. 37700339 10.1186/s13045-023-01498-2 PMC10498649

[20] V. Kansal , B. L. C. Kinney , and N. C. Schmitt , “Characterization of the Tumor Microenvironment in the Mouse Oral Cancer (MOC1) Model After Orthotopic Implantation in the Buccal Mucosa,” Head & Neck 46, no. 5 (2024): 1056–1062. 38445546 10.1002/hed.27722 PMC11003840

[21] A. P. Minz , D. Mohapatra , M. Dutta , et al., “Statins Abrogate Gemcitabine‐Induced PD‐L1 Expression in Pancreatic Cancer‐Associated Fibroblasts and Cancer Cells With Improved Therapeutic Outcome,” Cancer Immunology, Immunotherapy 72, no. 12 (2023): 4261–4278. 37926727 10.1007/s00262-023-03562-9 PMC10992415

[22] G. H. Nam , M. Kwon , H. Jung , et al., “Statin‐Mediated Inhibition of RAS Prenylation Activates ER Stress to Enhance the Immunogenicity of KRAS Mutant Cancer,” Journal for Immunotherapy of Cancer 9, no. 7 (2021): e002474. 34330763 10.1136/jitc-2021-002474 PMC8327837

[23] M. Kwon , G. H. Nam , H. Jung , et al., “Statin in Combination With Cisplatin Makes Favorable Tumor‐Immune Microenvironment for Immunotherapy of Head and Neck Squamous Cell Carcinoma,” Cancer Letters 522 (2021): 198–210. 34571082 10.1016/j.canlet.2021.09.029

[24] X. Ma , E. Bi , Y. Lu , et al., “Cholesterol Induces CD8(+) T Cell Exhaustion in the Tumor Microenvironment,” Cell Metabolism 30, no. 1 (2019): 143–156. 31031094 10.1016/j.cmet.2019.04.002 PMC7061417

[25] Y. Chen , Y. Zhu , X. Li , et al., “Cholesterol Inhibits TCR Signaling by Directly Restricting TCR‐CD3 Core Tunnel Motility,” Molecular Cell 82, no. 7 (2022): 1278–1287. 35271814 10.1016/j.molcel.2022.02.017

[26] X. Liu , X. Bao , M. Hu , et al., “Inhibition of PCSK9 Potentiates Immune Checkpoint Therapy for Cancer,” Nature 588, no. 7839 (2020): 693–698. 33177715 10.1038/s41586-020-2911-7 PMC7770056

[27] I. Okoye , A. Namdar , L. Xu , N. Crux , and S. Elahi , “Atorvastatin Downregulates Co‐Inhibitory Receptor Expression by Targeting Ras‐Activated mTOR Signalling,” Oncotarget 8, no. 58 (2017): 98215–98232. 29228684 10.18632/oncotarget.21003 PMC5716724

[28] M. Swamy , K. Beck‐Garcia , E. Beck‐Garcia , et al., “A Cholesterol‐Based Allostery Model of T Cell Receptor Phosphorylation,” Immunity 44, no. 5 (2016): 1091–1101. 27192576 10.1016/j.immuni.2016.04.011

[29] S. Tenesaca , M. Vasquez , M. Alvarez , et al., “Statins Act as Transient Type I Interferon Inhibitors to Enable the Antitumor Activity of Modified Vaccinia Ankara Viral Vectors,” Journal for Immunotherapy of Cancer 9, no. 7 (2021): e001587. 34321273 10.1136/jitc-2020-001587 PMC8320251

[30] Y. Wang , S. You , S. Su , et al., “Cholesterol‐Lowering Intervention Decreases mTOR Complex 2 Signaling and Enhances Antitumor Immunity,” Clinical Cancer Research 28, no. 2 (2022): 414–424. 34728526 10.1158/1078-0432.CCR-21-1535 PMC8776603

[31] Y. Xia , Y. Xie , Z. Yu , et al., “The Mevalonate Pathway Is a Druggable Target for Vaccine Adjuvant Discovery,” Cell 175, no. 4 (2018): 1059–1073. 30270039 10.1016/j.cell.2018.08.070

[32] W. Yang , Y. Bai , Y. Xiong , et al., “Potentiating the Antitumour Response of CD8(+) T Cells by Modulating Cholesterol Metabolism,” Nature 531, no. 7596 (2016): 651–655. 26982734 10.1038/nature17412 PMC4851431

[33] K. R. Getz , E. Bellile , K. R. Zarins , et al., “The Association Between Inflammatory Biomarkers and Statin Use Among Patients With Head and Neck Squamous Cell Carcinoma,” Head & Neck 44, no. 6 (2022): 1393–1403. 35338544 10.1002/hed.27040 PMC9088158

[34] Z. Yuan , M. O'Melia , K. Li , et al., “Tumor Microenvironment Impaired T Cell Antigen Recognition and Function Were Restored by Lovastatin Therapy,” bioRxiv 13 (2022): 507496.

